# Barriers to female leadership in intensive care medicine: insights from an ESICM NEXT & Diversity Monitoring Group Survey

**DOI:** 10.1186/s13613-024-01358-3

**Published:** 2024-08-19

**Authors:** Silvia De Rosa, Stefan J. Schaller, Laura Galarza, Ricard Ferrer, Bairbre A. McNicholas, Max Bell, Julie Helms, Elie Azoulay, Antoine Vieillard-Baron, the NEXT Committee & Diversity Monitoring Group of the ESICM Society

**Affiliations:** 1 Centre for Medical Sciences, CISMed, University of Trento, Via S. Maria Maddalena 1, 38122 Trento, Italy; 2 Anesthesia and Intensive Care, Santa Chiara Regional Hospital, APSS Trento, Trento, Italy; 3 Department of Anesthesiology, and Intensive Care Medicine (CCM/CVK), Charité, Universitätsmedizin Berlin, corporate member of Freie Universität Berlin, Humboldt-Universität Zu Berlin, and Berlin Institute of Health, Berlin, Germany; 4 School of Medicine and Health, Department of Anesthesiology and Intensive Care Medicine, Technical University of Munich, Munich, Germany; 5 Department of Intensive Care, Hospital General Universitari de Castelló, Castelló de la Plana, Spain; 6 SODIR Research Group, Intensive Care Department, Vall d’Hebron University Hospital, Vall d’Hebron Institut de Recerca, 08035 Barcelona, Spain; 7 Nephrology Services, Galway University Hospitals, SAOLTA University Healthcare Group, Galway, Ireland; 8 Department of Perioperative Medicine and Intensive Care, Karolinska University Hospital, Stockholm, Sweden; 9 Service de Médecine Intensive-Réanimation, Nouvel Hôpital Civil, Hôpitaux Universitaires de Strasbourg, 1, Place de l’Hôpital, 67091 Strasbourg Cedex, France; 10 Intensive Care Department, Medical Intensive Care Unit, APHP, Hôpital Saint Louis, Paris Cité University, Paris, France; 11 Service de Médecine Intensive Réanimation, Assistance Publique-Hôpitaux de Paris, University Hospital Ambroise Paré, 92100 Boulogne-Billancourt, France; 12 Department of Physiology and Pharmacology, Karolinska Institutet, Stockholm, Sweden

**Keywords:** Female leadership, Intensive care medicine, Gender equity, Workplace barriers, Professional development, Diversity and inclusion, Women’s leadership programs, Mentoring initiatives, Career advancement

## Abstract

**Background:**

The underrepresentation of women in leadership remains a pervasive issue, prompting a critical examination of support mechanisms within professional settings. Previous studies have identified challenges women face, ranging from limited visibility to barriers to career advancement. This survey aims to investigate perceptions regarding the effectiveness of women’s leadership programs, mentoring initiatives, and a specialized communication course. Particularly it specifically targets addressing the challenges encountered by professional women.

**Methods:**

This multi-center, observational, international online survey was developed in partnership between ESICM NEXT and the ESICM Diversity and Inclusiveness Monitoring Group for Healthcare. Invitations to participate were distributed to both females and men through emails and social networks. Data were collected from April 1, 2023, through October 1, 2023.

**Results:**

Out of 354 respondents, 90 were men (25.42%) and 264 were women (74.58%). Among them, 251 completed the survey, shedding light on the persistent challenges faced by women in leadership roles, with 10%-50% of respondents holding such positions. Women’s assertiveness is viewed differently, with 65% recognizing barriers such as harassment. Nearly half of the respondent’s experience interruptions in meetings. Only 47.4% receiving conference invitations, with just over half accepting them. A mere 12% spoke at ESICM conferences in the last three years, receiving limited support from directors and colleagues, indicating varied obstacles for female professionals. Encouraging family participation, reducing fees, providing childcare, and offering economic support can enhance conference involvement. Despite 55% applying for ESICM positions, barriers like mobbing, harassment, lack of financial support, childcare, and language barriers were reported. Only 14% had access to paid family leave, while 32% benefited from subsidized childcare. Participation in the Effective Communication Course on Career Advancement Goals and engagement in women’s leadership and mentoring programs could offer valuable insights and growth opportunities. Collaborating with Human Resources and leadership allies is crucial for overcoming barriers and promoting women’s career growth.

**Conclusions:**

The urgency of addressing identified barriers to female leadership in intensive care medicine is underscored by the survey’s comprehensive insights. A multifaceted and intersectional approach, considering sexism, structural barriers, and targeted strategies, is essential.

**Supplementary Information:**

The online version contains supplementary material available at 10.1186/s13613-024-01358-3.

## Introduction

There exists a notable sex imbalance in leadership positions within the field of intensive care medicine, presenting a multifaceted challenge that demands proactive measures to ensure the continued provision of high-quality and sustainable leadership in our specialty [1].

Targeted initiatives must be implemented to foster diversity and ensure the equitable representation of women in the field. Indeed, women are often not considered for leadership roles more than being explicitly told they are not ready. This reflects a form of gender bias that undermines women’s confidence and readiness to pursue leadership positions [2]. Additionally, women’s self-censorship, stemming from the internalization of societal messages about their capabilities, contributes to this issue. Research has identified a “confidence gap” where women tend to underestimate their abilities and hesitate to apply for leadership roles, perpetuating a cycle of underrepresentation in leadership [3]. At the same time, their male counterparts proactively pursue growth opportunities [4]. This results in women being considered for leadership positions only after they have meticulously fulfilled all prerequisites, often finding themselves overqualified for the roles they are offered. Despite the abundance of qualified women capable of assuming leadership roles, a significant gender gap persists in attaining the most lucrative and prestigious positions [5]. The prevailing definition of leadership qualities remains entrenched in an antiquated male model, perpetuating the exclusion of women from these roles. Despite considerable investments in women’s leadership programs over the past decades, progress in promoting women to leadership positions has reached a standstill [6]. Acknowledging the urgency of addressing and eliminating the gender leadership gap, the European Society of Intensive Care Medicine (ESICM) is committed to taking decisive action. To this end, the ESICM NEXT Committee and The Diversity and Inclusiveness Monitoring Group have collaboratively developed an observational survey. This survey extends to all health professionals engaged in critical care, primarily identifying, and understanding the barriers to female leadership. These barriers encompass perpetuated stereotypes, limited professional connections, bias and discrimination, and a lack of flexibility in leadership opportunities.

Through the administration of this survey, ESICM NEXT and the Diversity and Inclusiveness Monitoring Group aim to gather anonymous insights into how our work and personal environments influence our overall well-being, with a specific focus on identifying and addressing barriers faced by women in professional settings, such as gender discrimination, lack of access to leadership opportunities, and systemic biases. The objective is to formulate an extensive collection of pragmatic suggestions aimed at dismantling the obstacles impeding the advancement of female leaders in critical care. Through this endeavor, the initiative endeavors to foster a leadership landscape within the field that is more inclusive and equitable.

## Methods

This is an observational, international online open survey developed in partnership between ESICM NEXT, representing young intensivists < 38 years old within ESICM, and the Diversity and Inclusiveness Monitoring Group for Healthcare Professionals. The study was approved by the ethics committee of the University of Trento, Trento (2023-023), in accordance with the principles outlined in the Declaration of Helsinki. We obtained informed consent from all participants enrolled in the study at the beginning of the survey.

The questionnaire was distributed by the ESICM Society among its members, with a maximum of two reminders. In addition, invitations to participate were distributed through social networks (a link for email registration was disseminated via social media (i.e., Twitter, Facebook, and Linkedin). A concise survey introduction and corresponding link were crafted for sharing on social media platforms. The online questionnaire [Supplementary material 1] was available from April 1, 2023, through October 1, 2023 and preliminary results were reported AT Lives in Milan [7].

### Survey development

The objective of this survey was to gain insights into the opinions of healthcare professionals regarding three main aspects related to barriers to female leadership: (1) Sexism, (2) Structural Barriers, and (3) Strategies to Address the Challenges. We combined these topics in a single questionnaire with three sections to minimize the burden for respondents, as both topics are closely related and would be studied in the same target population.

The questionnaire was developed by a focus group (Silvia De Rosa, Stefan J. Schaller, Laura Galarza, Antoine Vieillard-Baron) of ESICM representatives. The development of the questionnaire involved an informal iterative process. Initially drafted based on key issues identified by the steering committee, the questionnaire was subsequently circulated among committee members for feedback. This collaborative approach, involving multiple rounds of review and refinement, ensured the clarity and relevance of the question.

The questionnaire was created using SurveyMonkey Platinum (SurveyMonkey Inc., San Mateo, CA, USA) with 32 questions. All answers could be reviewed and edited until final submission. Information about the survey, its purpose, and informed consent was explained at the beginning of the online survey.

Respondents’ demographics were collected in questions 1 to 8. The first section of the questionnaire (questions 9 to 15) focused on the definition of sexism (including sexual harassment, inequitable work environments, and subtler forms of sexism). The second section of the questionnaire (questions 16 to 28) included questions on structural barriers, specifically limited access to established networks. The third section of the questionnaire (questions 29 to 32) addressed strategies to address the challenges.

### Target population

Healthcare professionals working in critical care settings were the target population. In addition, they had the opportunity to contact the research team if they had additional questions or wanted to receive a summary of the study findings.

### Data analysis

Data were downloaded as a Comma-Separated Values (CSV) file, and stored as an Excel file (Microsoft Corp, Redmond, WA, USA). Responses were included in the analyses if participants answered the demographic questions and at least one question from the second section of the questionnaire. Exclusion criteria encompassed open-ended questions that were responded to in a language different from the intended language of the survey. Missing data were not imputed. Observations with missing data were considered as non-responses and were included in the overall analysis, acknowledging participants’ choice to skip certain questions. Statistics, conducted using MS Excel, were utilized to analyze the findings. Descriptive summaries for continuous variables employed the mean and standard deviation, while categorical variables were expressed through counts and percentages.

## Results

### Survey respondents

**Fig. 1:**
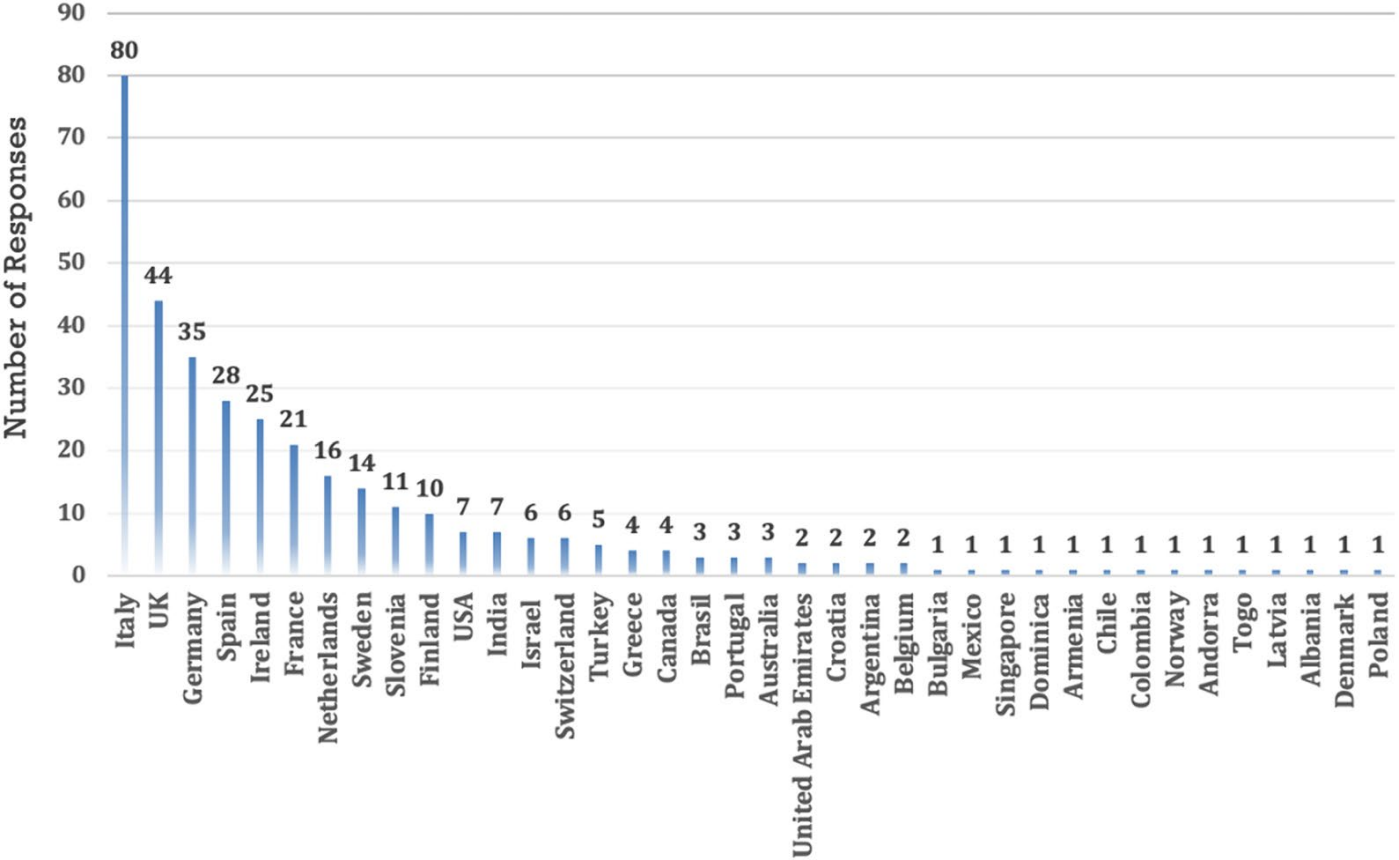
Geographical Distribution of Survey Participants Across Countries

Out of a total of 354 respondents who participated in the online survey, 251 completed the entire survey by answering all the questions and leaving no fields blank. However, it is worth noting that we also considered non-responses as a choice not to answer that question, thus still constituting a form of response. This means approximately 70.90% of the initial participants completed the entire survey. This completion rate indicates the attrition or dropout rate of participants as they progress through the survey. The demographic and professional characteristics are detailed in Table 1. Figure 1 depicts the Geographical Distribution of Survey Participants Across Countries. The majority, comprising individuals aged < 37 years (39.5%) and females (74.0%), contributed to the diverse respondent pool. Among them, 137 (54.58%) were ICU consultants, 66 (26.29%) were residents, 37 (14.74%) were ICU nurses/advanced clinical practitioners, 30 (11.95%) were heads of department/full professors, and 26 (10.36%) were associate professors. Most respondents identified as White/Caucasian (83.6%) and reported 10–20 years of healthcare experience (36.4%). Survey compilation primarily involved specialists with 10–20 years of healthcare experience (36.4%). Respondents were affiliated with various hospital types, with 44.9% associated with universities.

**Table 1:** Demographic and professional characteristics of survey participants

Variable	Total
	n = 354
Age, n (%)	
< 37	140 (39.5)
38–45	110 (31.1)
46–65	100 (28.2)
> 65	4 (1.13)
Sex, n (%)	
Female	262 (74.0)
Male	90 (25.4)
Other	2 (0.56)
Ethnicity, n (%)	
American Indian or Alaskan Native	1 (0.3)
Black or African American	4 (1.13)
Hispanic or Latino	24 (6.8)
White / Caucasian	296 (83.6)
Asian	17 (4.80)
Other	3 (0.85)
No answer	1 (0.28)
Years of experience in Healthcare, n (%)	
< 5	69 (19.5)
05-Sep	70 (19.8)
Oct-20	129(36.4)
> 20	86 (24.3)
Profession, n (%)	
ICU Nurse/Advanced Clinical Practitioner	37 (10.4)
ICU Respiratory Therapist	7 (1.98)
Medical Student	3 (0.85)
Resident	66 (18.6)
ICU Fellow	16 (4.52)
Doctoral student / PhD candidate	4 (1.13)
ICU consultant	137 (38.7)
Associate professor	26 (7.34)
Head of department/Full professor	30 (8.47)
Other	25 (7.06)
Hirsch index (mean ± SD)	**8.34 (± 11.3)**
Type of Hospital	
University	159 (44.9)
Teaching hospital of a university	125 (35.3)
Non-university public hospital	53 (15)
Private institution	17 (4.8)

Supplementary Table 1 and Table 2 shows respectively Gender Distribution and High-Respondent Countries.

### Sexism: gender bias, discrimination and stereotyping

**Fig. 2:**
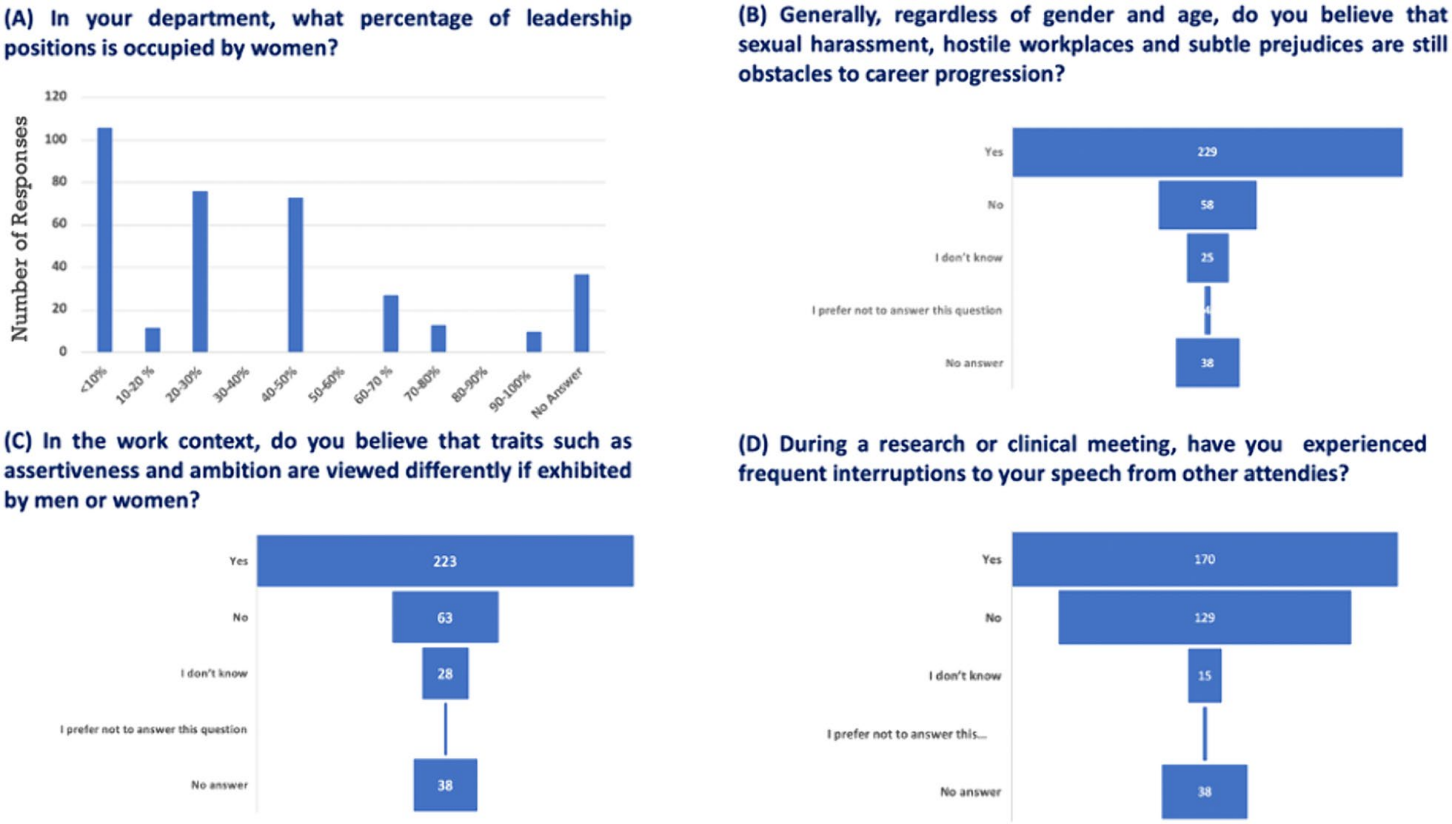
Perceived Gender Bias and Harassment. Respondents’ perceptions of gender bias, discrimination, and harassment, emphasizing differences in assertiveness and the impact on career advancement

**Fig. 3:**
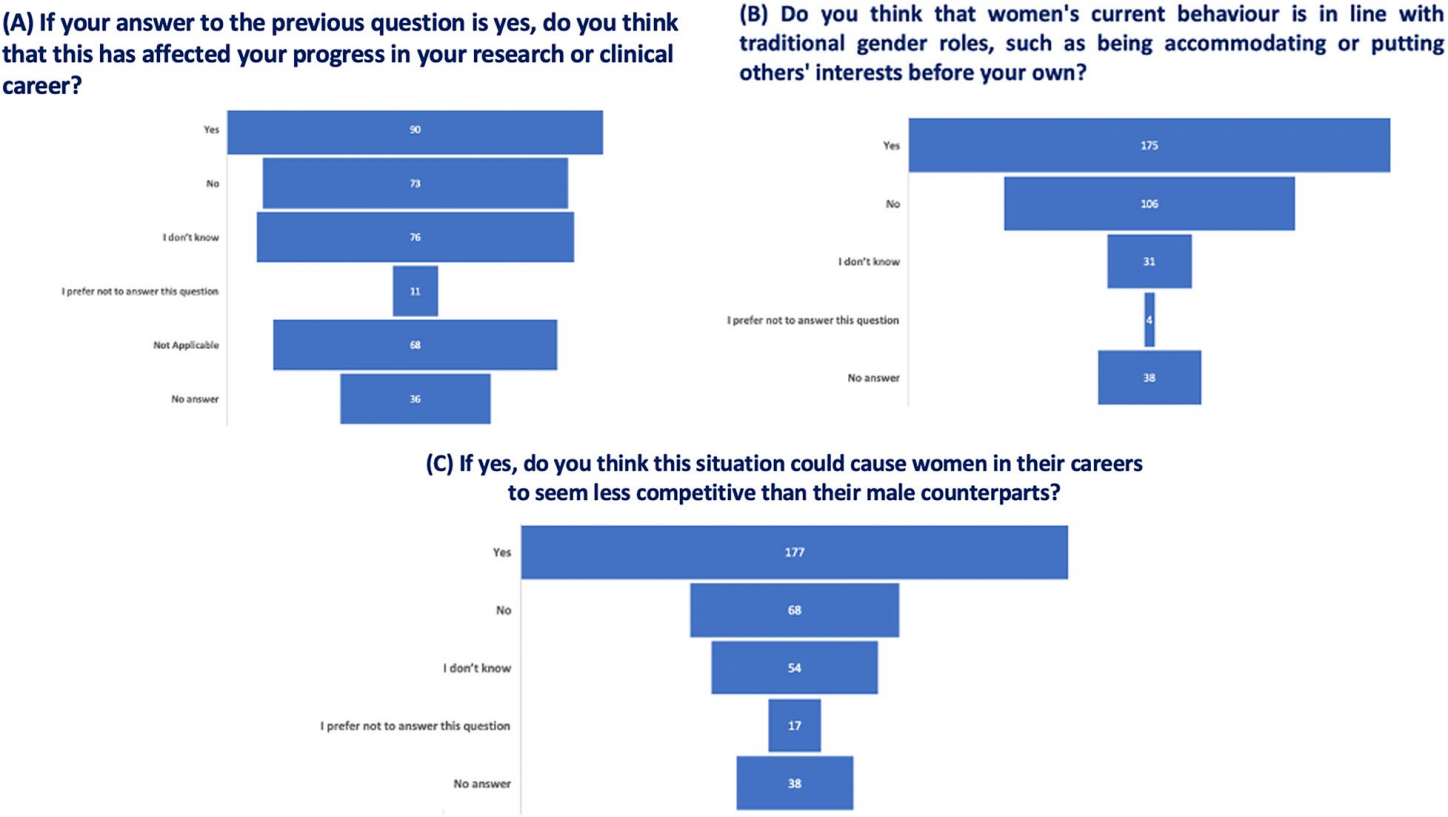
Impact of Traditional Gender Roles. Correlation between adherence to traditional gender roles and perceived competitiveness for career women

The reported range of female leadership positions in their respective countries, as indicated by women respondents, spans from 10 to 50%. Furthermore, the display of assertiveness, closely linked to ambition, is perceived differently when demonstrated by women (Fig. 2A and C). Regarding sexual harassment, 65% of respondents acknowledge that hostile work environments and subtle biases still serve as barriers to career advancement (Fig. 2B). Observable gestures, such as frequent interruptions during discussions as indicated by 48% of the responses (Fig. 2D), potentially impacting progress in research or clinical careers for approximately half of the respondents (see Fig. 3A). Women’s behavior appears to align with traditional gender roles, as indicated by 49% of the total respondents. Out of those who responded affirmatively, 50% believe that adhering to traditional gender roles may pose a risk to the perceived competitiveness of career women compared to their male counterparts (Fig. 3B and C).

### Structural barriers

#### Limited access to established networks

**Fig. 4:**
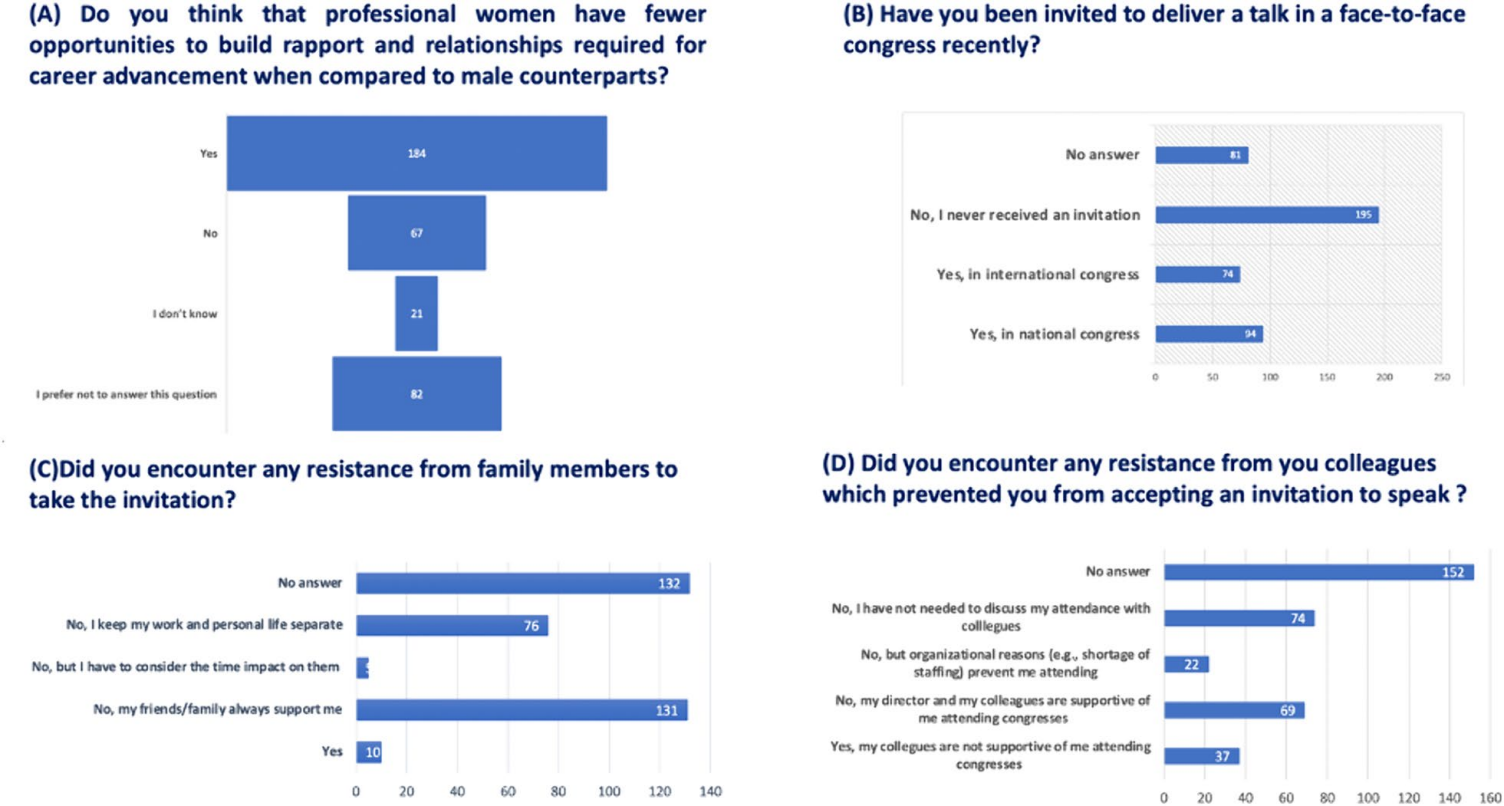
Barriers to Conference Participation. Barriers faced by respondents in participating in conferences, including invitations, acceptance rates, and reasons for refusal

Concerning the establishment of professional rapport by women in the field, a prevailing sentiment among respondents is the perception of fewer opportunities for professional women than their male counterparts (Fig. 4A). This perception highlights a significant structural barrier. Out of 184 respondents, 74 (40.2%) received invitations to international conferences, while 94 received invitations to national conferences. Of those invited to national meetings, 84 out of 96 accepted, whereas 71 out of 74 accepted invitations to international conferences. (Fig. 4B). Specifically, only 41 (11.6%) reported having been invited to speak at a face-to-face conference of the ESICM in the last three years, and of those, only 35 accepted the invitation. The reasons for refusal among 50 (14%) respondents are predominantly associated with issues such as lack of self-confidence, language barriers, inhibition, inadequacy, time management, maternity, absence of financial support, or unspecified reasons. These reasons underscore the structural barriers women face in accessing professional networks.

Although only 3% reported encountering resistance from family members, approximately 37% of respondents (131 individuals) assert that their friends and family consistently provide adequate support, while 21.47% (76 respondents) affirm that they deliberately maintain a separation between their private life and work (Fig. 4C). This lack of support from professional networks is a significant barrier.

In contrast, only 19.49% (69 respondents) reported receiving full support from their directors and colleagues to participate in conferences as speakers (Fig. 4D). Conversely, 6.21% (22 respondents) attribute their non-participation in conferences to organizational reasons, and 10.45% (37 respondents) report a lack of colleague support. These data points illustrate the lack of organizational support as a structural barrier.

In response to inquiries about considering an application for a position within the ESICM, 55% (195 respondents) responded affirmatively, but they encountered barriers such as workplace harassment (mobbing), language barriers, ethnic disparities, excessive workload, inconducive work environments, clinical service demands, family responsibilities, and a perceived lack of experience. These barriers further highlight the structural challenges in professional advancement.

Regarding strategies to facilitate attendance at conferences, respondents suggested measures such as economic support, facilitation for spousal presence, increased invitations for women, reduced opposition from colleagues, mitigation of gender disparities, provision of protected time, childcare support, implementation of a more structured departmental plan with hospital permission for participation, support programs, and family conciliation policies. These suggestions aim to address and overcome the structural barriers faced by women.

Fourteen percent (51 respondents) reported the availability of paid family leave in their country after becoming parents, while 62% (83 respondents) did not respond. Thirty-two percent (114 respondents) confirmed the existence of subsidized childcare in their country, whereas 24% (83 respondents) did not provide a response.

#### Strategies to address the challenges

**Fig. 5:**
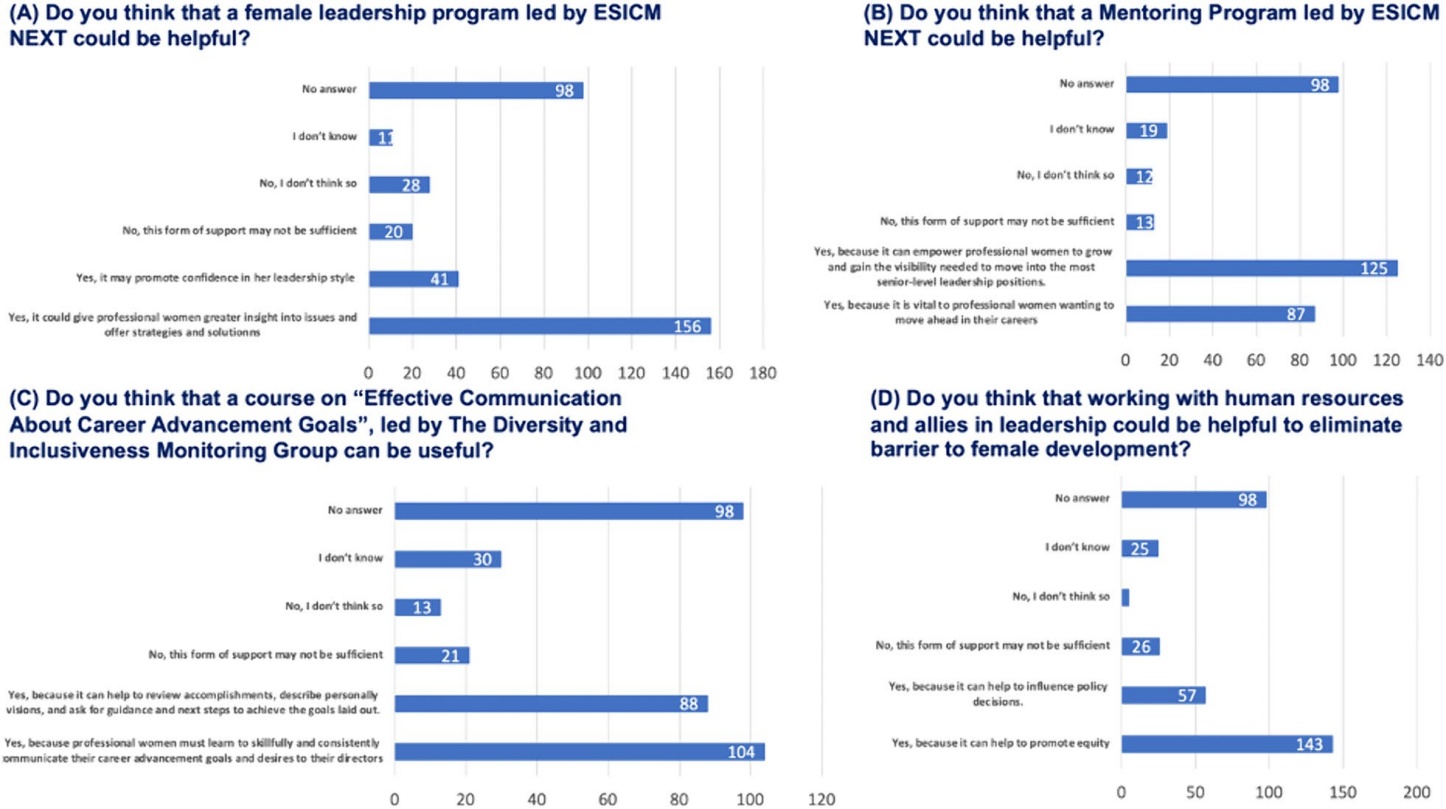
Effectiveness of Strategies. Demonstrates respondents’ opinions on the effectiveness of strategies such as women’s leadership programs, mentoring initiatives, communication courses, and collaboration with human resources

For 156 (44%) respondents, a women’s leadership program could provide professional women with greater insight into problems and offer strategies and solutions, and for 41 (12%) it may promote confidence in leadership style. Only a minority (20; 6%) responded that this form of support is not sufficient (Fig. 5A).

For 125 (35%) respondents, a mentoring program can allow professional women to grow and gain the visibility needed to move into the most senior level of leadership position; 87 (25%) believe that it can be vital to professional women wanting to advance in their careers. Only 13 (4%) individuals think this support may be insufficient (Fig. 5B).

A total of 104 (29%) responders affirms that an “Effective Communication on Career Advancement Goals Course,” conducted by the Diversity and Inclusion Monitoring Group, is necessary because professional women must learn to skillfully and consistently communicate their career advancement goals and desires to their directors. Eighty-eight (24%) respondents believe this course could help review accomplishments, describe personal visions, and seek guidance for the next steps to achieve their goals. Twenty-one respondents (6%) state this might be insufficient (Fig. 5C).

For the respondents, working with human resource departments and allies in leadership could help eliminate barriers to women’s development, enabling professional women to grow, gain visibility, and move forward in their careers because it can help to promote equity (143; 40%) and influence policy decisions (57; 16%). Twenty-six (7%) individuals assert that this support might not be sufficient (Fig. 5D).

## Discussion

### Main findings

Our survey highlights several key barriers to female leadership in intensive care medicine. First, the perception of fewer opportunities: a significant number of respondents (Fig. 4A) believe that professional women have fewer opportunities compared to their male counterparts. This perception reflects a critical structural barrier. Second, conference participation: only 41 respondents (11.6%) reported being invited to speak at an ESICM conference in the last three years, and of those, only 35 accepted the invitation. The reasons for refusal include lack of self-confidence, language barriers, and other structural issues (Fig. 4B). Third, support systems: approximately 37% of respondents received adequate support from family and friends, but only 19.49% received full support from their directors and colleagues (Fig. 4C and D). Fourth, barriers to ESICM positions: barriers such as workplace harassment, language barriers, and excessive workload were reported by 55% of respondents considering applications for ESICM positions. Fifth, strategies for improvement: respondents suggested measures such as economic support, facilitation for spousal presence, and childcare support to improve participation in professional activities.

### Importance of survey and addressing low response rate

The significance of this survey extends beyond identifying barriers to female leadership in intensive care medicine; it also serves as a critical tool for raising awareness about these pervasive issues within the professional community. However, an important concern that needs to be addressed is the low response rate, as only 354 out of an estimated 10,000 ESICM members participated in the survey. This low engagement might suggest a broader disengagement or perceived irrelevance of the topic among many potential respondents.

Possible factors include survey fatigue, lack of interest in the topic, or insufficient awareness of the survey’s distribution. More precise tracking mechanisms for distribution to ensure the survey reaches the intended audience effectively, as well as personalized invitations and targeted reminders that highlight the importance and impact of the survey, possono essere degli strumenti utili da poter usare in futuro. Additionally, providing incentives for participation and clearly communicating the potential benefits and significance of the survey results in influencing professional practices could encourage higher engagement.

Moreover, the low response rate itself highlights a significant barrier in terms of engagement and awareness about gender issues within the field. This underscores the need for ongoing efforts to sensitize and educate the professional community about the importance of addressing gender inequities. By fostering a culture that values and prioritizes gender equity, we can hope to see greater participation and engagement in future initiatives aimed at understanding and overcoming these barriers.

### Relationship with other studies

Sexual harassment, inequitable work environments, and subtler sexism impose significant challenges for professional women striving for success, notably evidenced in the inadequate representation within national intensive care medicine societies globally [1, 4, 5]. Venkatesh et al. [8] analyzed data from 2006 to 2017, revealing consistent under-representation of females in training programs, specialist positions, academic roles, and leadership within intensive care. These findings emphasize the imperative of targeted interventions and policy changes to cultivate a more inclusive environment for women in intensive care medicine. Additionally, Thompson et al. [9] in a cross-sectional survey highlighted gender disparities in healthcare perceptions, particularly in critical care, emphasizing differences in confidence regarding equity resolution, access to flexible work practices, and caregiver responsibilities. In 2019, the French Intensive Care Society initiated ten actions to promote gender equity, including mentorship, sponsorship, workshops on unconscious bias, and support for research on gender inequity [10]. Furthermore, a survey among French women intensivists revealed challenges such as work-life balance issues, gender-related career barriers, and experiences of discrimination and harassment, underscoring the need for flexible work arrangements and better support for professional and personal fulfillment [11]. Recent studies have further underscored the pervasive nature of gender biases affecting female professionals in intensive care medicine. For instance, Mehta et al. [12] reviewed women’s participation as faculty at five critical care conferences over seven years, highlighting that male speaker significantly outnumbered female speakers at all conferences. This study emphasized the need for systemic changes to address these disparities and promote gender equity in professional settings. Similarly, Dymore-Brown et al. [13] provided an update on gender disparity in critical care conferences, auditing scientific programs from 2017 to 2022. The study found an increased representation of female speakers and moderators over the years but noted a persistent gender gap, particularly among physicians. These findings highlight the importance of continued efforts to minimize gender inequalities and enhance support systems for women in critical care medicine.

### Online conferences

The transition to online conferences, accelerated by the COVID-19 pandemic, has had a notable impact on female participation in critical care conferences. Online platforms have reduced travel-related barriers, making it easier for women to balance professional responsibilities with personal and family commitments. According to recent data, the proportion of female speakers increased in online formats compared to traditional in-person conferences. For example, data from the Society of Critical Care Medicine showed that in 2021, when the conference was held online, 48% of the speakers were female, compared to an average of 27% In previous In-person years. Similarly, the State of the Art (SOA) conference achieved gender parity in 2022 with 48% female speakers when held online [12].

Online conferences also facilitate greater international participation, allowing women from diverse geographical locations to contribute without the logistical and financial constraints of travel. This inclusivity has broadened the representation of women in critical care, providing them with greater visibility and opportunities for networking and collaboration [13].

However, despite these advances, challenges remain. The data indicates a persistent gender gap, particularly among physician speakers. While the proportion of female moderators and speakers increased, the overall representation of female physicians at these conferences still lags their male counterparts. For instance, at the International Symposium on Intensive Care and Emergency Medicine (ISICEM), despite a statistically significant increase in female participation, male speakers continued to outnumber female speakers by a significant margin [12, 13].

### Female professional networks and success stories

The establishment of female professional networks has been instrumental in advancing gender equality in various specialties. Organizations such as Women in Critical Care Medicine (WICC) [14], International Women in Intensive and critical care (iWIN) [15], Women in Anesthesiology (WIA) [16] have created platforms for mentorship, networking, and professional development. These networks provide crucial support, enabling women to share experiences, access resources, and advocate for policy changes. Evidence from other specialties highlights successful initiatives that have achieved significant progress in gender equality. For example, the Association of Women Surgeons (AWS)[17] has been pivotal in promoting female leadership in surgery, leading to increased representation of women in top surgical positions. Similarly, the Women in Cardiology (WIC) [18] section of the American College of Cardiology has successfully implemented mentorship programs and leadership training, resulting in a higher number of women attaining leadership roles in cardiology. These success stories demonstrate the effectiveness of targeted interventions and the power of professional networks in breaking down barriers and fostering an inclusive environment.

### Sexism and gender bias

The high prevalence of sexual harassment within the workplace, hostile work environments, and subtle biases indicates a critical need for interventions to address workplace culture and eliminate discriminatory practices in intensive care medicine. The data also confirm that traditional gender roles continue to influence the perception of competitiveness for career women, emphasizing the importance of challenging and changing these ingrained norms. Recent findings by Tabassum and Nayak [19] indicate that descriptive and prescriptive gender stereotypes significantly impede women’s career advancement by reinforcing managerial and occupational biases in the workplace. Similarly, Ullrich et al. [20] highlighted the ongoing impact of traditional gender roles on women’s professional competitiveness, further supporting the need for targeted interventions to address these issues.

### Structural barriers: limited access to established networks

The survey illuminate’s disparities in opportunities for professional growth and visibility, particularly regarding invitations to conferences and speaking engagements. The observed barriers, such as self-confidence issues, language barriers, and inadequate support, highlight the multifaceted nature of challenges faced by women in their pursuit of leadership roles. The reported lack of support from colleagues and organizational reasons for non-participation in conferences indicate systemic issues that need targeted interventions. The findings also emphasize and support the importance of family support, organizational policies, and strategies to enhance work-life balance in fostering women’s career progression [21]. Nevertheless, health concerns have become a focal point in organizational literature. While our survey did not specifically evaluate this aspect, Gragnano et al. [22] propose the significance of work–health balance, challenging the notion that it is solely age-related and emphasizing its association with one’s health condition.

### Strategies to address the challenges

The identified strategies, including women’s leadership programs, mentoring initiatives, and communication courses, emerge as potential avenues to mitigate barriers and promote women’s advancement in intensive care medicine, as confirmed in the literature [23]. The positive perceptions of these strategies indicate recognition of their potential effectiveness. However, the acknowledgment that some respondents find these measures insufficient underscores the need for continuous refinement and expansion of support mechanisms. The crucial role of human resources and leadership allies in eliminating barriers is highlighted, emphasizing the importance of organizational culture and policies in fostering gender equity.

### A call for action: breaking down barriers for women in leadership

**Fig. 6:**
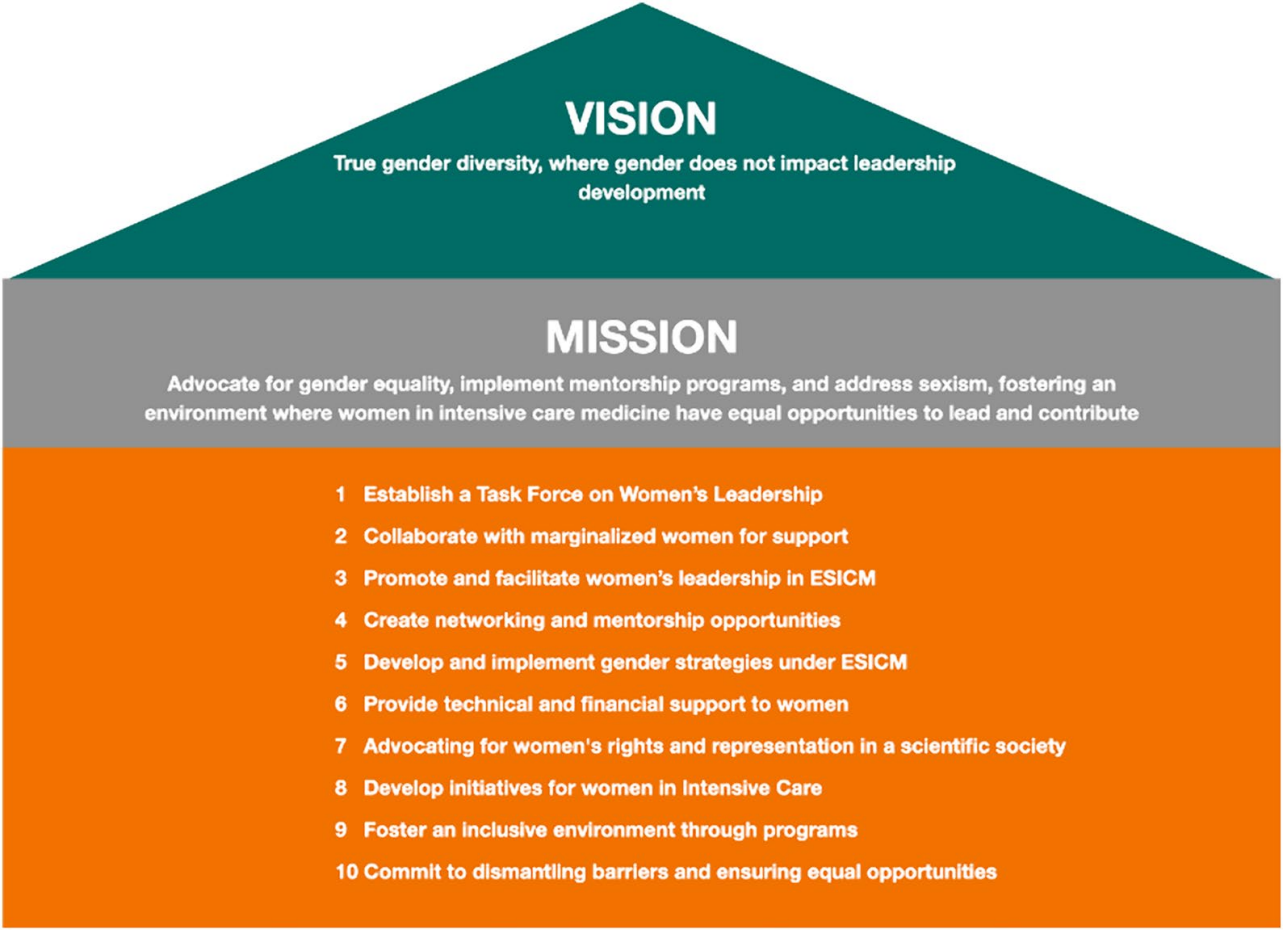
Empowering Women in Intensive Care: A Visionary Blueprint for Equality and Leadership

A compelling call to action is underway to dismantle barriers for women in leadership roles. This initiative involves creating a dedicated Task Force on Women’s Leadership (Fig. 6), focusing on collaboration with marginalized women to amplify their voices and provide crucial support. It encourages and supports women’s active participation and leadership within the ESICM society, fostering networks and mentorship opportunities. The strategy includes the establishment and implementation of gender-specific initiatives, aligned with a potential ESICM framework. Essential elements involve providing technical and financial support for women, advocating for their rights and representation, and developing inclusive programs for empowerment in intensive care for women. These measures are directly inspired by the recommendations of Hamzaoui et al. [10], who emphasized the importance of targeted interventions and policy changes to promote gender equity in intensive care medicine. Our findings are consistent with a broad range of literature that highlights similar challenges and proposed solutions in various medical specialties, indicating that this call for action could and must be generalized to other fields.

### Strengths and limitations of this study

Online surveys provide a means to collect anonymized data and streamline the data collection process. However, focusing specifically on professionals in intensive care medicine may limit the generalizability of the findings to other medical specialties or non-healthcare professions.

Nevertheless, our data align with extensive literature in other medical specialties, suggesting broader applicability. The survey was international, and the number of respondents varied between countries, which means the responses may reflect the situation in one country more than another, influenced by cultural factors. This variability is both a limitation and a strength, as it provides a diverse perspective while highlighting the need for context-specific strategies. Utilizing social media for survey distribution enables reaching healthcare personnel, but it introduces the possibility of selection bias, as individuals without a social media presence may be excluded. The survey was distributed through multiple channels, including direct emails to ESICM members and social media platforms. While it is estimated that the survey reached approximately 10,000 ESICM members based on the society’s membership database, the exact number of recipients cannot be precisely determined. This limitation makes it difficult to calculate an exact response rate. The survey’s response rate and sample representativeness were not explicitly outlined, posing a potential for response bias, particularly if participants with strong opinions or personal experiences were more inclined to respond, potentially influencing the outcomes. Additionally, we did not allocate space to address the aspect of work-life balance. In interpreting data regarding invitations to talks or leadership roles in a society like the ESICM, it must be acknowledged that approx. 40% were NEXT, i.e., young intensivists, and had a mean H-index of 8.3 (± 11.3) Therefore, most respondents will not have achieved international expert status yet, which will limit invitations and elections to society leadership positions outside NEXT. Furthermore, comparison data on male counterparts to identify differences are not available in our survey. An important limitation of our survey is that it only focused on barriers for females and did not investigate other components of inclusiveness or diversity.

## Conclusions

Intensive care medicine prides itself on its dynamic nature, dedication to continuous improvement, and understanding of the pivotal role of human factors in delivering effective patient care. However, despite this commitment, there exists a noticeable gender imbalance in leadership roles within the field. Measures are needed to secure the sustainability and quality of intensive care leadership, including cultural change, workplace structural reforms, advocacy, and mentoring.

### Supplementary Information

Additional file 1

## Data Availability

Data and Materials are available on reasonable request.
