# Supplementary material for: Barriers to female leadership in intensive care medicine: insights from an ESICM NEXT & Diversity Monitoring Group Survey
Source: Ann Intensive Care. 2024 Aug 19;14:126. doi: 10.1186/s13613-024-01358-3 (PMC11333654; doi:10.1186/s13613-024-01358-3)
Supplement: Supplementary file 1 — Additional file 1 [file 13613_2024_1358_MOESM1_ESM.docx]

# Supplementary Material

## Supplementary Table 1. Gender Distribution

| **QUESTION** | **ITA** | **UK** | **GER** | **SPN** | **IRE** | **FRAN** | **NETH** | **SE** | **SVN** | **FI** |
| --- | --- | --- | --- | --- | --- | --- | --- | --- | --- | --- |
| 1)In your department, what percentage of leadership positions is occupied by women?  <10%  10-20 %  20-30%  30-40%  40-50%  50-60%  60-70 %  70-80%  80-90%  90-100%  No Answer | 33 (41)  0 (0)  21 (26)  12 (15)  0 (0)  0 (0)  6 (8)  1 (1)  0 (0)  1 (1)  6 (8) | 14 (32)  0 (0)  9 (20)  0 (0)  9 (20)  0 (0)  2 (5)  0 (0)  0 (0)  6 (14)  4 (9) | 7 (20)  1 (2.9)  15(43)  0 (0)  8 (23)  0 (0)  0 (0)  0 (0)  0 (0)  0 (0)  4 (11) | 7 (25)  2 (7)  8 (29)  0 (0)  4 (14)  0 (0)  3 (11)  1 (4)  0 (0)  1 (4)  2 (7) | 6 (24)  0 (0)  3 (12)  0 (0)  4 (16)  0 (0)  5 (20)  2 (8)  0 (0)  2 (8)  4 (16) | 4 (25)  5 (31)  0 (0)  3 (19)  0 (0)  3 (19)  0 (0)  0 (0)  0 (0)  0 (0)  1 (6) | 5 (31)  0 (0)  4 (25)  0 (0)  4 (25)  0 (0)  1 (6)  0 (0)  0 (0)  0 (0)  1 (6) | 1 (7)  0 (7)  1 (7)  0 (0)  8 (57)  0 (0)  0 (0)  2 (14)  0 (0)  0 (0)  2 (14) | 1 (9)  0 (0)  2 (18)  0 (0)  5 (45)  0 (0)  0 (0)  0 (0)  0 (0)  0 (0)  1 (9) | 1 (10)  0 (0)  2 (20)  0 (0)  2 (20)  0 (0)  2 (20)  0 (0)  0 (0)  0 (0)  3 (30) |
| 2) Generally, regardless of gender and age, do you believe that sexual harassment, hostile workplaces and subtle prejudices are still obstacles to career progression?  Yes  No  I don’t know  I prefer not to answer this question  No answer | 56 (70)  13 (16)  5 (6)  0 (0)  6 (8) | 31 (70)  5 (11)  3 (7)  1 (2)  4 (9) | 25(71)  5 (14)  1 (2.9)  0 (0)  4 (11) | 18 (64)  6 (21)  1 (4)  1 (4)  2 (7) | 14 (56)  3 (12)  3 (12)  1 (4)  4 (16) | 12 (75)  2 (12)  1 (6)  0 (0)  1 (6) | 11 (69)  2 (12)  2 (12)  0 (0)  1 (6) | 8 (57)  4 (29)  0 (0)  0 (0)  2 (14) | 6 (54)  3 (27)  2 (18)  0 (0)  0 (0) | 4 (40)  2 (20)  1 (10)  0 (0)  3 (30) |
| 3) In the work context, do you believe that traits such as assertiveness and ambition are viewed differently if exhibited by men or women?  Yes  No  I don’t know  I prefer not to answer this question  No answer | 53 (66)  16 (20)  4 (5)  1 (1)  6 (8) | 26 (59)  7 (16)  7 (16)  0 (0)  4 (9) | 25(71)  5 (14)  1 (3)  0 (0)  4 (11) | 20 (71)  3 (11)  3 (11)  2 (7)  0 (0) | 11 (44)  6 (24)  4 (16)  0 (0)  4 (16) | 12 (75)  2 (12)  1 (6)  0 (0)  1 (6) | 9 (56)  4 (25)  2 (12)  1 (6)  0 (0) | 10 (71)  2 (14)  0 (0)  0 (0)  2 (14) | 6 (54)  4 (36)  1 (9)  0 (0)  0(0) | 3 (30)  3 (30)  4 (40)  3 (30)  0 (0) |
| 4) During a research or clinical meeting, have you experienced frequent interruptions to your speech from other attendees?  Yes  No  I don’t know  I prefer not to answer this question  No answer | 35 (44)  35 (44)  4 (5)  0 (0)  6 (8) | 25 (57)  11 (25)  3 (7)  1 (2)  4 (9) | 21(60)  10(29)  0 (0)  0 (0)  4 (11) | 15 (54)  9 (32)  1 (4)  1 (4)  2 (7) | 15 (60)  5 (20)  1 (4)  0 (0)  4 (16) | 7 (44)  6 (37)  2 (12)  0 (0)  1 (6) | 8 (50)  6 (37)  1 (6)  0 (0)  1 (6) | 4 (29)  7 (50)  1 (7)  0 (0)  2 (14) | 6 (54)  5 (45)  0 (0)  0 (0)  0 (0) | 2 (20)  5 (50)  0 (0)  0 (0)  3 (30) |
| 5) If your answer to the previous question is yes, do you think that this has affected your progress in your research or clinical career?  Yes  No  I don’t know  I prefer not to answer this question  Not Applicable  No answer | 20 (25)  23 (29)  11 (14)  3 (4)  17 (21)  6 (8) | 9 (20)  10 (23)  17 (39)  2 (5)  2 (5)  4 (9) | 8 (23)  4 (11)  12(34)  0 (0)  7 (20)  4 (11) | 9 (32)  6 (21)  3 (11)  8 (29)  0 (0)  2 (7) | 7 (28)  6 (24)  7 (28)  1 (4)  0 (0)  4 (16) | 7 (44)  1 (6)  1 (6)  0 (0)  0 (0)  1 (6) | 5 (31)  4 (25)  2 (12)  0 (0)  4 (25)  1 (6) | 2 (14)  1 (7)  3 (21)  0 (0)  6 (43)  2 (14) | 2 (18)  7 (64)  2 (18)  0 (0)  0 (0)  0 (0) | 1 (10)  1 (10)  3 (30)  2 (20)  0 (0)  3 (30) |
| 6) Do you think that women's current behaviour is in line with traditional gender roles, such as being accommodating or putting others' interests before your  own?  Yes  No  I don’t know  I prefer not to answer this question  No answer | 28 (35)  36 (45)  8 (10)  2 (3)  6 (8) | 32 (73)  6 (14)  1 (2)  1 (2)  4 (9) | 21(60)  7(20)  3(9)  0(0)  4(11) | 10 (36)  12 (43)  4 (14)  0 (0)  2 (7) | 16 (64)  2 (8)  2 (8)  1 (4)  4 (16) | 13 (81)  1 (6)  1 (6)  0 (0)  1 (6) | 10 (62)  4 (25)  1 (6)  0 (0)  1 (6) | 6 (43)  4 (29)  2 (14)  0 (0)  2 (14) | 3 (27)  7 (64)  1 (9)  0 (0)  0 (0) | 4 (40)  3 (30)  0 (0)  0 (0)  3 (30) |
| 7) If yes, do you think this situation could cause women in their careers to seem less competitive than their male counterparts?  Yes  No  I don’t know  I prefer not to answer this question  No answer | 31 (38.7)  23 (28.7)  15 (18.7)  5 (6.25)  6 (7.5) | 28 (64)  5 (11.)  5 (11)  2 (5)  4 (9) | 20(57)  4(11)  5(14)  2(6)  4(11) | 11(39)  6 (21)  8(29)  1(4)  2 (7) | 16 (64)  2 (8)  2 (8)  1 (4)  4 (16) | 13 (81)  1 (6)  1 (6)  0 (0)  1 (6) | 7 (44)  5 (31)  2 (13)  1 (6)  1 (6) | 9(64)  1(7)  1(7)  1(7)  2 (14) | 4 (36)  3 (27)  1 (9)  0 (0)  0 (0) | 4 (40)  0 (0)  1 (10)  2 (20)  3 (30) |
| 8) Do you think that professional women have fewer opportunities to build rapport and relationships required for career advancement when compared to male counterparts?  Yes  No  I don’t know  I prefer not to answer this question | 43 (53.7)  17 (21.2)  2 (2.5)  18 (22.5) | 18 (41)  3 (7)  8 (18)  15 (34) | 18(51)  8(23)  1(3)  7(20) | 16 (57)  4 (14)  9 (32)  5 (18) | 13 (52)  3 (12)  1 (4)  8 (32) | 14 (87)  1 (6)  0 (0)  1 (6) | 8 (50)  5 (31)  2 (12)  2 (12) | 7(50)  2 (14)  1(7)  1(7) | 2 (18)  6 (54)  1 (9)  2 (18) | 3 (30)  2 (20)  1 (20)  4 (40) |
| 9) Have you been invited to deliver a talk in a face-to-face congress recently?  Yes, in national congress  Yes, in international congress  No, I never received an invitation  No Answer | 22 (27.5)  10 (12.5)  30 (37.5)  18 (22.5) | 18 (41)  3 (7)  8 (18)  15(34) | 16(46)  6(17)  9(26)  6(17) | 10 (36)  4 (14)  9 (32)  5 (18) | 3 (12)  7 (28)  7 (28)  8 (32) | 6 (37)  5 (31)  4 (25)  1 (6) | 4 (25)  5 (31)  6 (37)  0 (0) | 2 (14)  3 (21)  5 (36)  4 (29) | 8 (73)  1 (9)  0 (0)  2 (18) | 4 (40)  2 (20)  0 (0)  4 (40) |
| 10) Did you encounter any resistance from family members to take the invitation?  Yes  No, my friends family always support me   No, but I have to consider the time impact on them  No, I keep my work and personal life separate  No answer | 1 (1.25)  36 (45)  0 (0)  16 (20)  27 (33.7) | 0 (0)  9 (20)  1 (2)  9 (20)  25 (57) | 2 (5.7)  9 (26)  0 (0)  13(37)  11(31) | 2 (7)  8 (29)  0 (0)  8 (29)  10 (36) | 1 (4)  7 (28)  0 (0)  6 (24)  11 (44) | 2 (13)  3 (19)  3 (19)  4 (25)  0 (0) | 0 (0)  12 (75)  0 (0)  1 (6)  3 (19) | 0 (0)  3 (21)  0 (0)  3 (21)  8 (57) | 2 (13)  3 (19)  3 (19)  4 (25)  0 (0) | 0 (0)  1 (10)  2 (20)  4 (40)  5 (50) |
| 11) Did you encounter any resistance from you colleagues which prevented you from accepting an invitation to speak ?  Yes, my colleagues are not supportive of me attending congresses  No, my director and my colleagues are supportive of me attending congresses  No, but organizational reasons (e.g., shortage of staffing) prevent me attending  No, I have not needed to discuss my attendance with colleagues  No answer | 9 (11.2)  16 (20)  6 (7.5)  18 (22.5)  31 (38.7) | 3 (9)  1(2)  2 (4)  9 (20)  29(66) | 3 (9)  9 (26)  4 (11)  6 (17)  13(37) | 4 (14)  11 (39)  1 (4)  1 (4)  11 (39) | 2 (8)  0 (0)  1 (4)  4 (16)  18 (72) | 2 (13)  4 (25)  2 (13)  4 (25)  4 (25) | 1 (6)  0 (0)  2 (13)  2 (13)  3 (19) | 0 (0)  4 (29)  0 (0)  2 (14)  8 (57) | 2 (13)  4 (25)  2 (13)  4 (25)  4 (25) | 0 (0)  0 (0)  3 (30)  0 (0)  7 (70) |
| 12) Do you think that a female leadership program led by ESICM NEXT could be helpful?  Yes, it could give professional women greater insight into issues and offer strategies and solutions  Yes, it may promote confidence in her leadership style  No, this form of support may not be sufficient  No, I don’t think so  I don’t know  No answer | 41 (51.2)  7 (8.8)  3 (3.8)  5 (6.3)  5 (6.3)  19 (25.7) | 17(39)  2(4)  2 (4)  4 (9)  1(2)  18(41) | 17(39)  2(4)  2 (4)  4 (9)  1(2)  18(41) | 11 (39)  2 (7)  3 (11)  4 (14)  1 (4)  7 (25) | 11 (44)  3 (12)  0 (0)  1 (4)  0 (0)  10 (40) | 5 (31)  4 (25)  0 (0)  3 (19)  1 (6)  3 (19) | 9 (56)  2 (13)  2 (13)  1 (6)  2 (13)  0 (0) | 5 (36)  2 (14)  1 (7)  1 (7)  1 (7)  4(29) | 5 (31)  4 (25)  0 (0)  2 (13)  2 (13)  3 (19) | 0 (0)  3 (30)  1 (10)  0 (0)  1 (10)  5 (50) |
| 13) Do you think that a Mentoring Program led by ESICM NEXT could be helpful?    Yes, because it is vital to professional women wanting to move ahead in their careers  Yes, because it can empower professional women to grow and gain the visibility needed to move into the most senior-level leadership positions.    No, this form of support may not be sufficient    No, I don’t think so    I don’t know  No answer | 25 (31.2)  25 (31.2)  2 (2.5)  3 (3.7)  6 (7.5)  0 (0) | 9 (20)  16 (36)  0 (0)  0 (0)  2 (5)  17 (39) | 12(34)  11(31)  1(3)  3(9)  1(3)  7 (20) | 4 (14)  13 (46)  1 (4)  2 (7)  0 (0)  8 (29) | 7 (28)  5 (20)  1 (4)  0 (0)  1 (4)  11 (44) | 4 (25)  5 (31)  0 (0)  2 (13)  2 (13)  3 (19) | 3 (18)  7 (44)  1 (6)  0 (0)  3 (19)  0 (0) | 1 (7)  6 (43)  1 (7)  1 (7)  1 (7)  4 (29) | 6 (38)  4 (25)  0 (0)  1 (6)  2 (13)  3 (19) | 0 (0)  3 (30)  1 (10)  1 (10)  0 (0)  5 (50) |
| 14) Do you think that a course on “Effective Communication About Career Advancement Goals”, led by The Diversity and Inclusiveness Monitoring Group can be useful?  Yes, because professional women must learn to skillfully and consistently communicate their career advancement goals and desires to their directors  Yes, because it can help to review accomplishments, describe personal visions, and ask for guidance and next steps to achieve the goals laid out.  No, this form of support may not be sufficient    No, I don’t think so  I don’t know  No answer | 32 (40)  19 (23.7)  1 (1.25)  4 (5)  5 (6.3)  19 (23.7) | 13 (29.5)  9 (20.5)  2 (4.5)  0 (0)  2 (4.5)  18 (40.9) | 15(43)  8 (23)  3 (9)  1 (3)  1 (3)  3 (9) | 4 (14)  8 (29)  8 (29)  3 (11)  2 (7)  7 (25) | 6 (24)  6 (24)  2 (8)  0 (0)  1 (4)  10 (40) | 6 (38)  4 (25)  0 (0)  1 (6)  2 (13)  3 (19) | 5 (31)  5 (31)  5 (31)  0 (0)  2 (12)  2 (12) | 2 (14)  4 (28)  0 (0)  1 (7)  3 (21)  4 (29) | 6 (38)  4 (25)  0 (0)  1 (6)  2 (13)  3 (19) | 0 (0)  2 (20)  1 (10)  1 (10)  1 (10)  5 (50) |
| 15) Do you think that working with human resources and allies in leadership could be helpful to eliminate barrier to female development?  Yes, because it can help to promote equity  Yes, because it can help to influence policy decisions.  No, this form of support may not be sufficient    No, I don’t think so  I don’t know  No answer | 41 (51.2)  11 (13.7)  3 (3.7)  0 (0)  6 (7.5)  19 (23.7) | 12 (27.3)  10 (22.7)  0 (0)  0 (0)  4 (9.1)  18 (41) | 18(51)  4 (11)  3 (9)  0 (0)  3 (9)  7 (20) | 9 (32)  8 (29)  0 (0)  3 (11)  1 (4)  7 (25) | 10 (40)  2 (8)  2 (8)  0 (0)  1 (4)  10 (40) | 7 (44)  3 (19)  1 (6)  0 (0)  2 (13)  3 (19) | 5 (31)  5 (31)  5 (31)  9 (56)  2 (12)  2 (12) | 5 (36)  2 (14)  1 (7)  0 (0)  2 (14)  4 (29) | 7(44)  3 (19)  1 (6)  0 (0)  2 (13)  3 (19) | 1 (10)  3 (30)  1 (10)  0 (0)  0 (0)  5 (50) |

Note: SE, Sweden; SVN, Slovenia;

## Supplementary Table 2. High-Respondent Countries.

| **QUESTION** | **Female** | **Male** | **Other** |
| --- | --- | --- | --- |
| 1)In your department, what percentage of leadership positions is occupied by women?  <10%  10-20 %  20-30%  30-40%  40-50%  50-60%  60-70 %  70-80%  80-90%  90-100%  No Answer | 90 (37.5)  10 (4.17)  53 (22.08)  49 (20.42)  0 (0)  18 (7.5)  13 (5.4)  0 (0)  0 (0)  7 (2.92)  22(9.17) | 16 (21.3)  2 (2.67)  23 (30.7)  24 (32.0)  0 (0)  8 (10.7)  0 (0)  0 (0)  0 (0)  2 (2.7)  15 (16.7) | 0 (0)  0 (0)  0 (0)  0 (0)  0 (0)  2 (100)  0 (0)  0 (1)  0 (0)  0 (0)  0 (0) |
| 2) Generally, regardless of gender and age, do you believe that sexual harassment, hostile workplaces and subtle prejudices are still obstacles to career progression?  Yes  No  I don’t know  I prefer not to answer this question  No answer | 194 (81)  29 (12.1)  13 (5.4)  4 (1.7)  22 (8.7) | 35 (46.7)  29 (38.7)  11 (14.7)  0 (0)  15 (16.7) | 0 (0)  0 (0)  2 (100)  0 (0)  0 (0) |
| 3) In the work context, do you believe that traits such as assertiveness and ambition are viewed differently if exhibited by men or women?  Yes  No  I don’t know  I prefer not to answer this question  No answer | 192 (80)  26 (10.8)  20 (8.3)  2 (0.8)  22 (8.7) | 31 (41.3)  37 (49.3)  7 (9.3)  0 (0)  15 (16.7) | 0 (0)  0 (0)  0 (0)  0 (0)  2 (100) |
| 4) During a research or clinical meeting, have you experienced frequent interruptions to your speech from other attendees?  Yes  No  I don’t know  I prefer not to answer this question  No answer | 118 (48.7)  104 (42.9)  18 (7.42)  2 (0.8)  22 (8.7) | 18 (24)  49 (65.3)  7 (9.3)  1 (1.3)  15 (16.7) | 1 (50)  1 (50)  0 (0)  0 (0)  0 (0) |
| 5) If your answer to the previous question is yes, do you think that this has affected your progress in your research or clinical career?  Yes  No  I don’t know  I prefer not to answer this question  Not Applicable  No answer | 61 (25.4)  137 (57.1)  20 (8.3)  22 (9.2)  0 (0)  22 (8.7) | 7 (9.3)  54 (72)  6 (8)  8 (10.7)  0 (0)  15(16.7) | 0 (0)  2 (100)  0 (0)  0 (0)  0 (0)  0 (0) |
| 6) Do you think that women's current behaviour is in line with traditional gender roles, such as being accommodating or putting others' interests before your  own?  Yes  No  I don’t know  I prefer not to answer this question  No answer | 129 (53.7)  63 (26.2)  28 ( 11.7)  21 (8.7)  22 (8.7) | 29 (38.7)  26 (34.7)  15 (20)  5 (6.67)  15 (16.7) | 1 (50)  1 (50)  0 (0)  0 (0)  0 (0) |
| 7) If yes, do you think this situation could cause women in their careers to seem less competitive than their male counterparts?  Yes  No  I don’t know  I prefer not to answer this question  No answer | 162 (67.5)  39 (16.2)  28 (11.7)  11 (4.6)  22 (8.7) | 33 (44)  27 (36)  12 (16)  3 (4)  15 (16.7) | 0 (0)  1 (50)  1 (50)  0 (0)  0 (0) |
| 8) Do you think that professional women have fewer opportunities to build rapport and relationships required for career advancement when compared to male counterparts?  Yes  No  I don’t know  I prefer not to answer this question  No answer | 155 (64.6)  50 (20.8)  21 (8.7)  14 (5.8)  22 (8.7) | 41 (54.7)  22 (29.3)  6 (8)  6 (8)  15 (16.7) | 2 (100)  0 (0)  0 (0)  0 (0)  0 (0) |
| 9) Have you been invited to deliver a talk in a face-to-face congress recently?  Yes, in national congress  Yes, in international congress  No, I never received an invitation  No Answer | 88 (36.7)  74 (30.8)  78 (32.5)  22 (9.17) | 28 (37.3)  29 (38.7)  18 (24)  15 (16.7) | 0 (0)  2 (100)  0 (0)  0 (0) |
| 10) Did you encounter any resistance from family members to take the invitation?  Yes  No, my friends family always support me   No, but I have to consider the time impact on them  No, I keep my work and personal life separate  No answer | 64 (26.7)  68 (28.3)  78 (32.5)  22 (9.2)  22 (8.8) | 8 (10.7)  20 (26.7)  18 (24)  15 (16.7)  15 (16.7) | 0 (0)  1 (50)  0 (0)  0 (0)  1 (50) |
| 11) Did you encounter any resistance from you colleagues which prevented you from accepting an invitation to speak ?  Yes, my colleagues are not supportive of me attending congresses  No, my director and my colleagues are supportive of me attending congresses  No, but organizational reasons (e.g., shortage of staffing) prevent me attending  No, I have not needed to discuss my attendance with colleagues  No answer | 30 (12.5)  144 (60)  36 (15)  19 (7.9)  22 (8.7) | 5(6.7)  57 (76)  5(6.7)  19 (7.9)  22 (8.7) | 0 (0)  2 (100)  0 (0)  0 (0)  0 (0) |
| 12) Do you think that a female leadership program led by ESICM NEXT could be helpful?  Yes, it could give professional women greater insight into issues and offer strategies and solutions  Yes, it may promote confidence in her leadership style  No, this form of support may not be sufficient  No, I don’t think so  I don’t know  No answer | 152 (58.3)  69 (28)  15 (6.2)  7 (2.9)  9 (3.7)  22 (8.7) | 41 (54.7)  22 (29.3)  6 (8)  3 (4)  3 (4)  15 (16.7) | 1 (50)  1 (50)  0 (0)  0 (0)  0 (0)  0 (0) |
| 13) Do you think that a Mentoring Program led by ESICM NEXT could be helpful?    Yes, because it is vital to professional women wanting to move ahead in their careers  Yes, because it can empower professional women to grow and gain the visibility needed to move into the most senior-level leadership positions.    No, this form of support may not be sufficient    No, I don’t think so    I don’t know  No answer | 140 (58.3)  69 (28)  15 (6.2)  7 (2.9)  9 (3.7)  22 (8.7) | 38 (50.67)  25 (33.33)  7 (9.33)  3 (4)  2 (2.7)  15 (16.67) | 1 (50)  0 (0)  1 (50)  0 (0)  0 (0)  0 (0) |
| 14) Do you think that a course on “Effective Communication About Career Advancement Goals”, led by The Diversity and Inclusiveness Monitoring Group can be useful?  Yes, because professional women must learn to skillfully and consistently communicate their career advancement goals and desires to their directors  Yes, because it can help to review accomplishments, describe personal visions, and ask for guidance and next steps to achieve the goals laid out.  No, this form of support may not be sufficient    No, I don’t think so  I don’t know  No answer | 124 (51.7)  83 (34.6)  11 (4.6)  6 (2.5)  11 (4.6)  22 (8.7) | 34 (45.3)  29 (38.7)  3 (4)  2 (2.7)  2 (2.7)  15 (16.7) | 1 (50)  0 (0)  1 (50)  0 (0)  0 (0)  0 (0) |
| 15) Do you think that working with human resources and allies in leadership could be helpful to eliminate barrier to female development?  Yes, because it can help to promote equity  Yes, because it can help to influence policy decisions.  No, this form of support may not be sufficient    No, I don’t think so  I don’t know  No answer | 112 (46.7)  53 (22.1)  37 (15.4)  13 (5.4)  18 (7.5)  22 (8.7) | 31 (41.3)  20 (26.7)  9 (12)  4 (5.3)  4 (5.3)  15 (16.7) | 1 (50)  0  1 (50)  0 (0)  0 (0)  0 (0) |

## Supplementary Material 3 – Original Survey

**Barriers to Female Leadership:**

**ESICM NEXT & The Diversity and Inclusiveness Monitoring Group Joint Survey**

Dear Colleague,

Despite efforts to achieve equity in the workplace, professional women face barriers to becoming recognized leaders.

There is no shortage of qualified women to fill leadership roles. Still, men are far more likely than women to take on the highest paying and prestigious leadership roles. The “qualities” of a leader are still largely based on an outdated male model that shuts women out.

Although decades of investment in women's leadership programs, progress in promoting women has stalled. ESICM would like to bridge the gender leadership gap once and for all.

For this reason, ESICM NEXT Committee and The Diversity and Inclusiveness Monitoring group designed an observational survey for all health professionals involved in critical care to assess barriers to female leadership: Old Stereotypes, Fewer Connections, Bias and Discrimination and Lack of Flexibility.

With this survey, ESICM NEXT and Diversity and Inclusiveness Monitoring Group want to anonymously explore how our wellbeing is impacted by our work and personal environments and develop a suite of practical recommendations for decrease barriers to Female Leadership

Sincerely,

**NEXT ESICM Committee**

**The Diversity and Inclusiveness Monitoring Group**

**Section I - General Information**

**What is your Age?**

- <37
- 38-45
- 46-65
- >65

**Which is your Gender?**

- Female
- Male
- Other

**Which is your Profession?**

- ICU Nurse/Advanced Clinical Practitioner
- ICU Respiratory Therapist
- Medical Student
- Resident
- ICU Fellow
- Doctoral student / PhD candidate
- ICU consultant
- Associate professor
- Head of department/Full professor
- Other (please, specify): _______

**Years of experience in Healthcare**

- - <5
  - 5-9
  - 10-20
  - >20

**Your H-index**

- - Google Scholar:

**In what country do you work?: (list of all countries) ___**

**What is your ethnicity?**

- American Indian or Alaskan Native
- Black or African American
- Hispanic or Latino
- White / Caucasian
- Asian
- Other (please specify): _____

**Type of Hospital:**

- University
- Teaching hospital of a university
- Non-university public hospital
- Private institution

**Section II – Sexism**

Sexual harassment, inequitable work environments, and subtler forms of sexism place a huge burden on professional women working toward their goals.

**Gender Bias, Discrimination and Stereotyping**

In your department, what percentage of leadership positions is occupied by women?

- <10%
- 10-20 %
- 20-30%
- 30-40%
- 40-50%
- 50-60%
- 60-70 %
- 70-80%
- 80-90%
- 90-100%

Generally, regardless of gender and age, do you believe that sexual harassment, hostile workplaces, and subtle prejudices are still obstacles to career progression?

- Yes
- No
- I don’t know
- I prefer not to answer this question

In the work context, do you believe that traits such as assertiveness and ambition are viewed differently if exhibited by men or women?

- Yes
- No
- I don’t know
- I prefer not to answer this question

During a research or clinic meeting, have you experienced frequent interruptions to your speech from other attendees?

- Yes
- No
- I don’t know
- I prefer not to answer this question

if your answer to the previous question is yes, do you think that this has affected your progress in your research or clinical career?

- Yes
- No
- I don’t know
- I prefer not to answer this question
- Not applicable

Do you think that women's current behaviour is in line with traditional gender roles, such as being accommodating or putting others' interests before your own?

- Yes
- No
- I don’t know
- I prefer not to answer this question

If Yes, do you think that in this way the career woman risks appearing less competitive than her male counterparts?

- Yes
- No
- I don’t know
- I prefer not to answer this question

**Section III – Structural Barriers**

**Limited Access to Established Networks**

Do you think that professional women have fewer opportunities to build rapport and relationships required for career advancement when compared to male counterparts?

- Yes
- No
- I don’t know
- I prefer not to answer this question

Have you been invited to deliver a talk in a face-to-face congress recently?

- Yes, in national congress
- Yes, in international congress
- No, I never received an invitation

If yes, did you accept the invitation?

- Yes
- No

Have you been invited to deliver a talk in a face-to-face congress of the ESICM in the last 3 years (LIVES, LIVES Forum, LIVES Asia,…)

- Yes
- No

If yes, did you accept the invitation?

- Yes
- No

If no, which is the reason (open question)?

Did you encounter any resistance from family members to take the invitation?

- Yes
- No, my frineds/family always support me
- No, but I have to consider the time impact on them
- No, I keep my work and personal life separate

Did you encounter any resistance from you colleagues which prevented you from accepting an invitation to speak? (choose all that apply)

- Yes, my collegues are not supportive of me attending congresses
- No, my director and my colleagues are supportive of me attending congresses
- No, but organizational reasons (e.g., shortage of staffing) prevent me attending
- No, I have not needed to discuss my attendance with colllegues

What could help facilitate your attendance at congresses? (open question)

Have your ever considered applying for a position in the ESICM?

- Yes
- No

If yes, did you encounter any barriers? (open question)

In your country, do you have the possibility to Paid Family Leave after becoming parents?

- Yes
- No

In your country, do you have a subsidized Child Care?

- Yes
- No

**Section IV Strategies to Address the Challenges**

Do you think that a female leadership program led by ESICM NEXT could be helpful?

- Yes, it could give professional women greater insight into issues and offer strategies and solutions
- Yes, it may promote confidence in her leadership style
- No, this form of support may not be sufficient
- No, I don’t think so
- I don’t know

Do you think that a Mentoring Program led by ESICM NEXT could be helpful?

- Yes, because it is vital to professional women wanting to move ahead in their careers
- Yes, because it can empower professional women to grow and gain the visibility needed to move into the most senior-level leadership positions.
- No, this form of support may not be sufficient
- No, I don’t think so
- I don’t know

Do you think that a course on “Effective Communication About Career Advancement Goals”, led by The Diversity and Inclusiveness Monitoring Group can be useful?

- Yes, because professional women must learn to skillfully and consistently communicate their career advancement goals and desires to their directors
- Yes, because it can help to review accomplishments, describe personal visions, and ask for guidance and next steps to achieve the goals laid out.
- No, this form of support may not be sufficient
- No, I don’t think so
- I don’t know

Do you think that working with human resources and allies in leadership could be helpful to eliminate barrier to female development?

- Yes, because it can help to promote equity
- Yes, because it can help to influence policy decisions.
- No, this form of support may not be sufficient
- No, I don’t think so
- I don’t know
